# Global-and-Local Context Network for Semantic Segmentation of Street View Images

**DOI:** 10.3390/s20102907

**Published:** 2020-05-21

**Authors:** Chih-Yang Lin, Yi-Cheng Chiu, Hui-Fuang Ng, Timothy K. Shih, Kuan-Hung Lin

**Affiliations:** 1 Department of Electrical Engineering, Yuan Ze University, Taoyuan 32003, Taiwan; andrewlin@saturn.yzu.edu.tw; 2 Department of Computer Science & Information Engineering, National Central University, Taoyuan City 32001, Taiwan; chialinwu001@gmail.com (Y.-C.C.); paul92035@gmail.com (K.-H.L.); 3 Department of Computer Science, University Tunku Abdul Rahman, Kampar 31900, Malaysia

**Keywords:** semantic segmentation, global context, local context, fully convolutional networks

## Abstract

Semantic segmentation of street view images is an important step in scene understanding for autonomous vehicle systems. Recent works have made significant progress in pixel-level labeling using Fully Convolutional Network (FCN) framework and local multi-scale context information. Rich global context information is also essential in the segmentation process. However, a systematic way to utilize both global and local contextual information in a single network has not been fully investigated. In this paper, we propose a global-and-local network architecture (GLNet) which incorporates global spatial information and dense local multi-scale context information to model the relationship between objects in a scene, thus reducing segmentation errors. A channel attention module is designed to further refine the segmentation results using low-level features from the feature map. Experimental results demonstrate that our proposed GLNet achieves 80.8% test accuracy on the Cityscapes test dataset, comparing favorably with existing state-of-the-art methods.

## 1. Introduction

**Figure 1:**
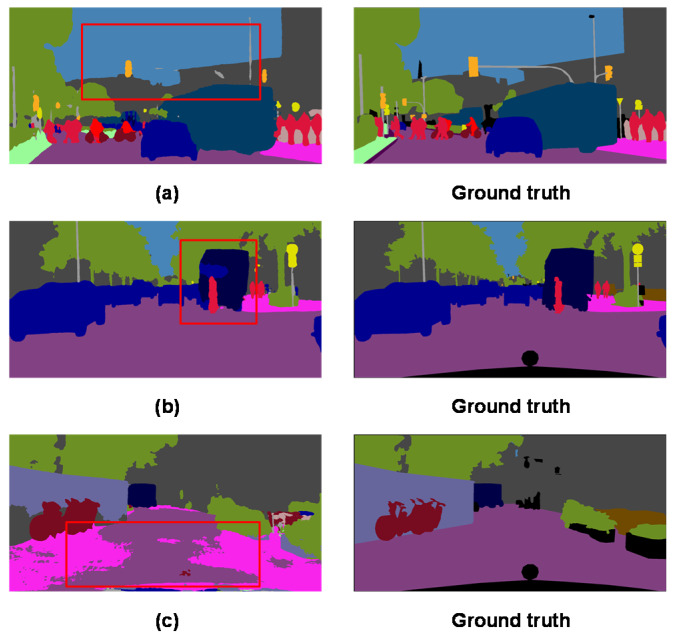
Some semantic segmentation issues observed in the Cityscapes dataset. (**a**) Missing small and thin objects. (**b**) Incorrect segmentation of similar parts. (**c**) Incomplete segmentation of large objects.

Image semantic segmentation is a computer vision task in which each pixel of an image is assigned to a corresponding object label. Semantic segmentation provides richer information, including object’s boundary and shape, than other object segmentation techniques such as object detection which produces only object bounding boxes. Therefore, semantic segmentation of street view images is important to the development of vision sensors for autonomous vehicle systems that require rich information for scene understanding [1,2]. Many state-of-the-art semantic segmentation methods are based on fully convolutional networks (FCN) [3]. FCN is an end-to-end, pixel-wise fully convolutional neural network for image semantic segmentation which replaces the fully connected layer in a conventional convolutional neural network (CNN) with convolutional layers and then restores the output to the original image size by up-sampling the final convolutional layer using a deconvolution operation [4]. However, consecutive pooling operations at the encoding stage usually lead to the reduction of feature resolution, which in turn will degrade final segmentation performance. As shown in the first row of Figure 1, small objects such as poles and traffic light are missing due to loss of detailed information resulted from pooling operations. To address this issue, *dilated* convolution (also called *atrous* convolution) has been proposed to replace the down-sampling and up-sampling operations in order to gain a wider field of view while preserving full spatial information [5,6,7].

Local context information is an important component in semantic segmentation for segmenting complex street view scenes that contain different objects with similar features. The second row in Figure 1 shows a portion of the ‘truck’ object that has been mistakenly classified as a ‘car’ object because it has a similar appearance to cars. Consequently, local context information should be explicitly encoded in the semantic segmentation models [6]. Another important factor affecting segmentation is the existence of objects in multiple scales. With regard to this, different scales of an image are generated to extract multi-scale features, and the features are then merged to predict the results [8,9,10]. In [11] and [12], dilated convolution layers with different rates were used to capture object features at multiple scales, and the experimental results indicated that using multiple field-of-views can improve overall segmentation accuracy.

Convolutional operations process one local neighborhood at a time as in a conventional CNN [13,14], which may cause incomplete segmentation of large objects. As shown in the third row of Figure 1, with local convolutional operations, parts of the road near roadside area were wrongly predicted as sidewalk due to the lack of global context information. To resolve this, global contextual information has been utilized in scene segmentation and been shown to be effective in reducing false segmentation [15]. However, a systematic way to integrate both global and local contextual information in a single network has not yet been fully investigated.

**Figure 2:**
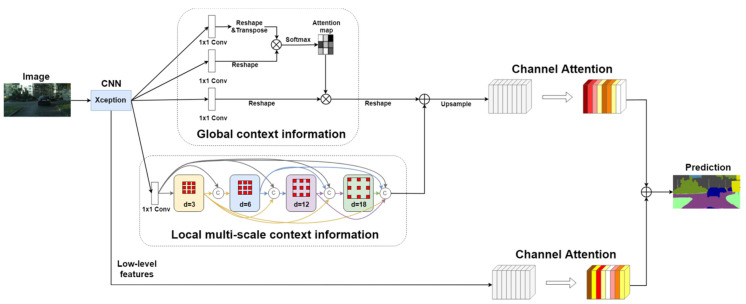
An overview of the proposed GLNet. First, a CNN is applied on the input image and the feature map from the last convolution layer of CNN is kept. Next, global module and local module are deployed on the feature map to capture both global and multi-scale context information. Finally, the channel attention module restores edges and fine details of the segmented objects using low-level features.

In this paper, we propose a global-and-local context network (GLNet) for semantic segmentation of street view images for autonomous vehicle systems, which incorporates both global and local contextual information in a single network. An overview of the proposed GLNet is shown in Figure 2. The global context module captures semantic of spatial interdependencies while the local context module uses dilated convolution of different rates to extract dense multi-scale features that are important for a network to adapt to different object sizes. GLNet fuses the outputs of the two modules to capture both global and local contextual information to help the network recognize the relationships between objects in a scene, helping eliminate false alarms. Furthermore, CNN low-level features are used through channel weighting to restore the edges and fine details of the segmentation results.

## 2. Related Work

Many recent image semantic segmentation methods have been based on fully convolutional networks (FCN) [3], which train on an end-to-end network for pixel-wise prediction, achieving state-of-the-art result. For instances, encoder-decoder networks such as SegNet [16] and U-Net [17] have been proposed to enhance semantic segmentation results. The encoder gradually reduces the spatial dimension of feature maps to extract salient features. On the other hand, the decoder increasingly up-samples the feature map back to the original spatial resolution of the input image and produces pixel-wise segmentation result. However, the encoding process usually causes the reduction of feature resolution, which in turn degrades final segmentation performance.

The use of contextual information is important to pixel-level prediction tasks. Objects in complex scenes exhibit large-scale changes, which introduces great challenges for advanced feature representations. In [11] and [12], dilated convolutions were used to expand the receptive field and to encode multi-scale context information. Zhao et al. [18] proposed a pyramid scene parsing network to capture multi-scale context information by aggregating feature maps of different resolutions through a pyramid pooling module. DeepLabv3+ [19] used parallel Atrous Spatial Pyramid Pooling (ASPP) which connects parallel dilated convolutions of different rates on the feature map to effectively encode multi-scale information. Zhou et al. [20] designed a multi-scale deep context convolutional network for semantic segmentation which combines the feature maps from different levels of network. Although multi-scale context information help to capture different scales objects, it cannot leverage the relationship between objects in a global view, which is also essential to scene segmentation.

DANet [21] uses self-attention to capture long-range global context by exploring orthogonal relationships in both spatial and channel dimensions using non-local operator [15]. Convolution is essentially a local operation. Non-local operations extract long-range dependencies directly from any two positions in an image. The position attention module of DANet selectively aggregates the features of each location by weighted summation of all locations. CFNet [22] adds an extra global average pooling path to determine whole scene statistics. However, both models do not incorporate local multi-scale features in their networks. Self-attention is also used in OCNet [23] to learn pixel-level object context information to enhance context aggregation. Attempt to incorporate additional depth information for improving semantic segmentation has also been explored in [24] and [25]. Our proposed method incorporates rich global spatial information and dense local multi-scale context information to model the relationship between objects in a scene to reduce segmentation errors.

## 3. Method

In this section, we describe the proposed GLNet network in detail. The overall network structure of GLNet is shown in Figure 2. First, an Xception [26] network with 65 layers is used as the backbone to extract features from the input image. Next, the feature map from the last convolution layer of the Xception network is input into both the global module and the local module and the outputs from the two modules are fused to generate the segmentation result. Lastly, the channel attention module refines the segmentation result by utilizing the low-level features from the Xception network.

### 3.1. Global Module

**Figure 3:**
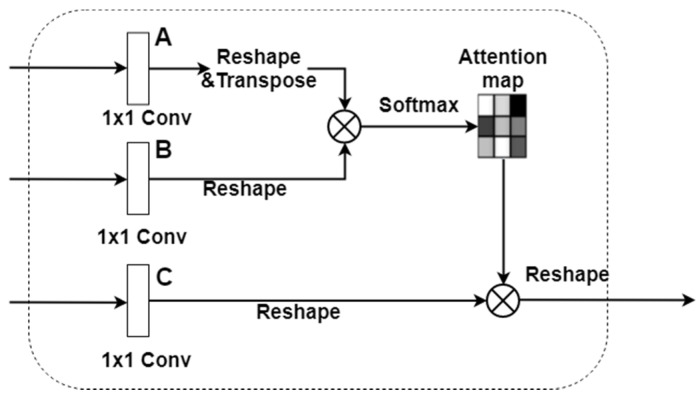
The global module. The number of channels of the CNN feature map is first reduced to 1/8 of the input channels before generating the attention map, and the weighted result is reverted back to the shape of the input feature map.

The global module is intended to capture the spatial dependencies of the feature map. To this end, we have adopted the non-local operations proposed in [15] and the details of the global module are shown in Figure 3. In the global module, the response at a feature location is weighted by features at all locations in the input feature map. The weights are determined based on the feature similarity between two corresponding locations. Thus, any two positions with similar features can contribute to each other regardless of the distance between them.

The last layer of the Xception network is first convolved with a 1 × 1 convolution to reduce the number of channels from 2048 to 256, or 1/8 of the original number of channels. This produces a *H* × *W* × *nc* feature map, where *H*, *W* are the height and width of the feature map, and *nc* denotes the number of channels. Three copies of the feature map (**A**, **B**, **C**) are generated and reshaped to *N* × *nc*, where *N* = *H* × *W*. As shown in Figure 3, feature map **A** is then transposed and multiplied by feature map **B**, and the resulting *N* × *N* matrix is passed through softmax function to produce the spatial attention map. Finally, feature map **C** is multiplied by the attention map and the result is reshaped back to *H* × *W* × *nc*. As a result, the output of the global module is a feature map weighted by the global spatial interdependencies among pixel locations.

### 3.2. Local Module

Objects in a scene usually exhibit large-scale changes, posing a difficult challenge for feature representation in semantic segmentation since multi-scale information must be properly encoded. DeepLab [19] handled this problem using an Atrous Spatial Pyramid Pooling (ASPP) module in which dilated convolutions with different rates are applied to the input feature map and the results are fused to account for different object scales. However, the ASPP module is not dense enough to deal with large object scale changes in complex scenes.

**Figure 4:**
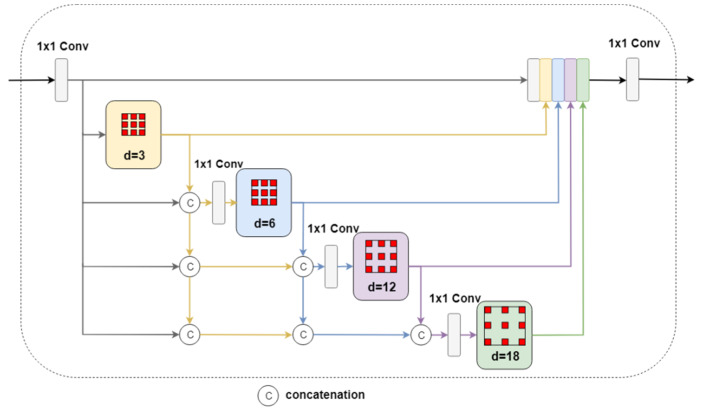
The local module. The output of each dilated convolutional layer is concatenated with the input feature map and convolved with 1 × 1 convolution to maintain fixed channel number before going into the next dilated convolution layer.

To resolve this, we propose a local module based on DenseASPP [12] to extract dense multi-scale features that are important for a network to adapt to large-scale variations. The local module combines the advantages of parallel and cascaded dilated convolutions to obtain larger and denser receptive field than ASPP, thus achieving superior multi-scale representation of objects in a scene. The details of the local module are depicted in Figure 4. Similar to the global module, the feature map from the Xception network is first convolved with 1 × 1 convolution to reduce the number of channels to *nc* (1/8 of the input channels) before it is fed to the local module. Next, the input feature map is sequentially convolved with dilated convolution with increasing dilation rates from 3, 6, 12, to 18. To lessen network complexity, dilation rate 24 is not included here as in DenseASPP. The input to each dilated convolution layer is formed by concatenating the input feature map with the outputs from previous convolutions and then convolved with 1 × 1 convolution to maintain the number of channels at *nc*. Lastly, the outputs from the four dilated convolutions are concatenated together with the input feature map and result is again reduced to *nc* channels before outputting to the next processing step. Compared with DenseASPP, the total number of parameters of the local module is reduced from 6.48 M to 2.01 M. It can be seen from Figure 4 that the output of the local module is a feature map that contains dense multi-scale feature information for the input image.

### 3.3. Channel Attention Module

**Figure 5:**
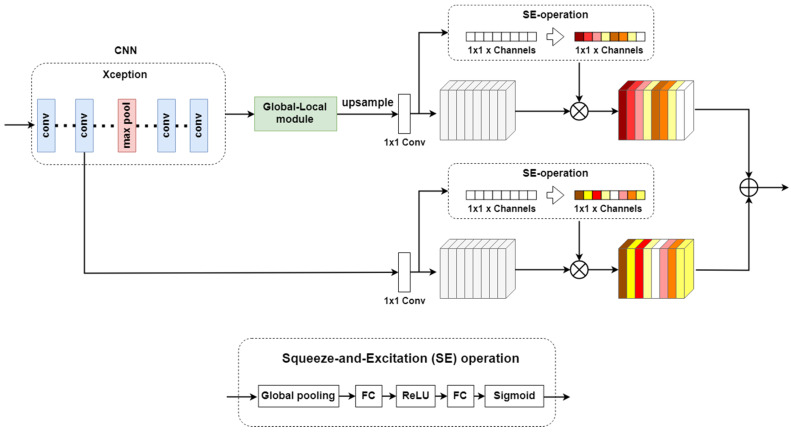
The Channel attention module. The output of the Global-Local module is up-sampled to match the size of the CNN feature map before the fusion process, and the SE operation explores channel dependency in the feature map.

The Global-Local module extracts high level multi-scale semantic information. However, details of objects may be lost during the down sampling process in the initial stage of the CNN network. The low-level feature maps produced by CNN before the max pooling layer should preserve detailed information including edges and other fine details of the objects in the image. To recover the finer details of objects, we propose a channel attention module which fuses the output from the Global-Local module and the low-level feature map of CNN, as shown in Figure 5.

The output of the Global-Local module is up-sampled to match the dimension of the low-level feature map of CNN before the fusion process. In addition, Squeeze-and-Excitation (SE) block [27] is applied to both feature maps to obtain cross channel information and learn the channel-wise attention. The SE block generates a weight between 0 and 1 per channel through sigmoid function, and the weights are then used to perform dynamic feature reweighting via channel-wise multiplication between the weights and the feature maps. As a result, the channel attention module also explores channel dependency in the feature map, which has been proven useful for image classification and segmentation tasks [28].

## 4. Experiments

In this section, we describe the implementation details and discuss the experimental results of the proposed GLNet network. Comprehensive experiments and ablation studies on the Cityscapes dataset [29] were carried out to evaluate the proposed network, and the performance improvements of the modules are highlighted.

### 4.1. Implementation Details

The implementation of GLNet is built upon the TensorFlow framework. The pre-trained weights of Xception-65 [26] were drawn from the Imagenet [30]. The original image size was 1024 × 2048, while the input image size was randomly cropped to 768 × 768. We adopted the learning rate policy from [6,18] where the current learning rate is calculated as follows:

(1) $${\left(1-\frac{iter}{\max\_iter}\right)}^{power},$$

where *iter* and *max_iter* represent the current training step and the overall training iterations, respectively. An initial learning rate of 0.01 was used throughout the experiments, whereas *max_iter* was set at 90,000 steps and *power* is set to 0.9. Furthermore, the batch size was fixed at 8 in our experiments.

The loss function is defined as:

(2) $$\mathrm{loss}=\sum_{n=1}^{N}l_{mce}\left(\left(X_{n}\right),Y_{n}\right),$$

where *X_n_* are the training images and *Y_n_* are the corresponding ground truth, and *l_mce_* denotes the multi-class cross-entropy loss for predictions.

The training data came from the Cityscapes dataset, which is comprised of 5000 annotated urban street scenes (2975 for training, 500 for validation and 1525 for testing) for pixel-level semantic labeling. Intersection over union (IoU) and pixel accuracy averaged across the 19 classes in the dataset were used as performance metrics for evaluating the methods. The GLNet takes about 6 h of training time using a single NVIDIA Tesla^®^ V100 GPU with batch size fixed at 8.

### 4.2. Ablation Study

An extensive ablation study on the Cityscapes dataset is conducted to determine the effectiveness of the various modules and the design choice of the proposed GLNet.

#### 4.2.1. Effect of Global Context Information

**Figure 6:**
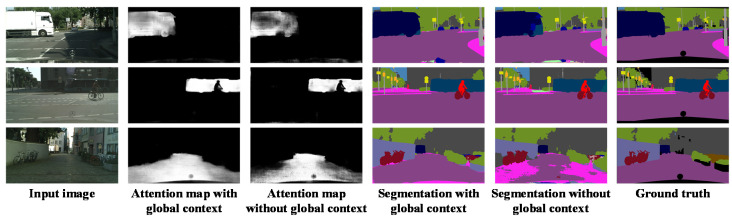
Visualization results of incorporating global context information versus without global context information.

Before examining the overall performance of the proposed GLNet network, we first investigate the effect of incorporating global context information on semantic segmentation, which is one of the main objectives of this study. Figure 6 shows the sample segmentation results when incorporating global context information versus results without the global context information. The first column is the input images. The second column shows visualizations of the attention maps generated by the Global-Local module, which are the results of fusing the outputs from both the global and the local modules (see Figure 2). The third column shows the same attention map, excluding the output from the global module.

It can be seen in Figure 6 that the attention maps that incorporated global context information are less noisy than those without global context information. The segmentation results in column 4 and 5 confirm that without the global context information, it is more problematic to segment larger objects, such as the truck in the first row, the bus in the second row, and the road in the last row. This indicates that using information from local context alone and ignoring the global scene context will result in misclassified pixels.

#### 4.2.2. GLNet Modules

**Figure 7:**
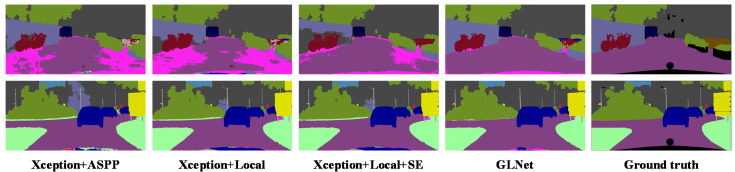
Visualization results on Cityscapes val. set. Refer to Table 1 for descriptions of corresponding modules.

In this experiment, we tested the effects of the various modules in GLNet on the overall performance of the network. The results are summarized in Table 1 and the visualization results are shown in Figure 7. The first test was to compare the proposed local module with the ASPP module used in DeepLabv3+ [19]. The local module increased the mean IoU by 0.5% over the ASPP module on the Cityscapes dataset. The first and second columns in Figure 7 show a slight decrease in false classification of pixels using the proposed local module. The above results suggest that dense multi-scale features extracted by the local module have positive influence on the overall segmentation results.

Next, we tested the effect of applying the channel attention module on the segmentation performance, which fuses low-level feature using SE block. With the inclusion of SE block, the mean IoU increased about 1%, from 77.6% to78.4%. Finally, when all three modules in GLNet were activated, the mean IoU improved to 80.1%, 3% higher than using only the ASPP module of DeepLabv3+ [19]. By visually examining the segmentation results in Figure 7, we can see that the proposed GLNet architecture has significantly fewer segmentation errors, especially for large objects and for the background area. This is due to the inclusion of global information in the feature extraction process.

#### 4.2.3. Global Module

As mentioned in Section 3, non-local block [15] was chosen to gather global attention in the proposed global module. It is worth to experiment with other attention mechanisms for capturing global attention in the feature map. The Convolutional Block Attention Module (CBAM) proposed recently by Woo et al. [31] and the Pyramid Pooling Module in PSPNet [18] are selected here to compare with the non-local block method. CBAM applies average pooling and max pooling along the channel axis of the feature map and then the result is passed through a 7 × 7 convolution to capture both cross-channel and spatial attentions [31]. Pyramid pooling module harvests different sub-region representations and concatenates them into a feature representation that carries both local and global context information [18].

**Figure 8:**
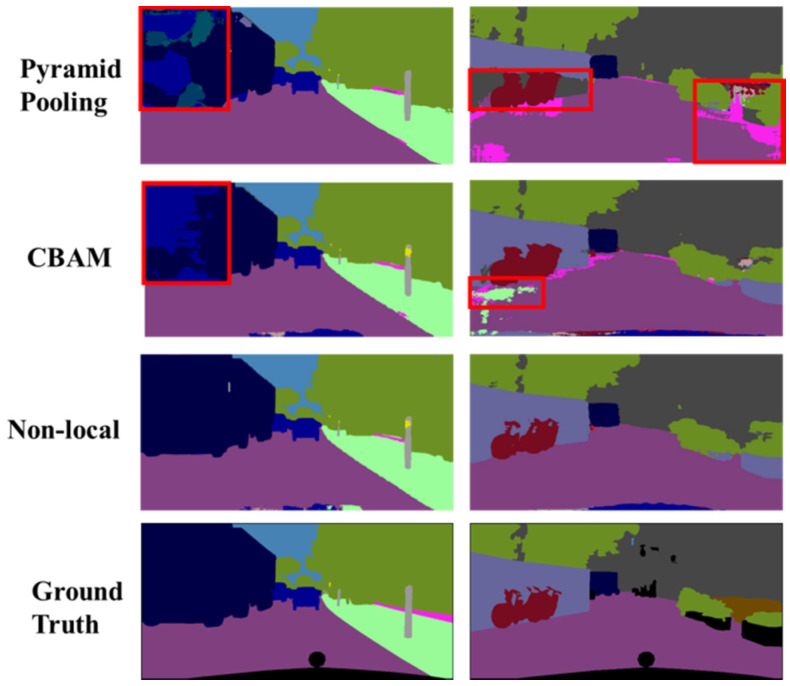
Visualization results on Cityscapes val. set of applying different methods in the global module.

The results of applying different methods for extracting global context information in the global module are shown in Table 2. The results indicate that using non-local block offers a slight edge over CBAM on the Cityscapes dataset. Pyramid pooling does not perform as well and has the lowest IoU score. One possible explanation of the low performance for pyramid pooling is that in the original PSPNet implementation, the number of output channels after concatenation is 3072, which has been reduced to 256 channels in order to fit our network architecture. The reduction in channel number might result in loss of feature information. Visualization results in Figure 8 confirm that non-local block can handle large objects better than CBAM and pyramid pooling. For example, as shown in the upper left column in Figure 8, CBAM and pyramid pooling misclassified portion of the truck object as car pixels, whereas non-local block was able to segment the truck object correctly. Therefore, non-local block is recommended to be used in the proposed GLNet network.

#### 4.2.4. Fusion vs. Concatenation

Another experiment was to explore the use of concatenation operation versus fusion operation for combining high-level feature and low-level feature and the results are shown in Table 3. The results demonstrate that the performance of using fusion operation is slightly better than using a concatenation operation. In-depth analysis of the segmentation results of individual object classes reveal that concatenation operation actually produced slightly better segmentation of small objects and fine details, thus providing nicer visualization results. Conversely, fusion operation generated better segmentation results on larger objects such as buses and trucks. Since large objects occupy more pixels and contribute more to the IoU calculation, as a result, the mean IoU under fusion operation is higher.

### 4.3. Performance Comparison

#### 4.3.1. Performance on Cityscapes Dataset

We have benchmarked our proposed GLNet with state-of-the-art methods on the Cityscapes test set and validation set and the results are shown in Table 4 and Table 5. All methods are trained with the fine dataset of Cityscapes. For the test set, GLNet achieves a mean IoU of 80.8, the highest among all the methods tested. Out of the 19 classes in the dataset, GLNet obtains or shares the best scores for 13 classes. Similar results are reported on the validation set, where GLNet has the best overall segmentation performance. Other methods, for example PSPNet [18], extract context information through global average pooling, and concatenates the information in the final output feature map. Although such an approach produces satisfactory segmentation results, but some fine details are missing and it generates false edges in the results. The works in [11,12,19] use Atrous Spatial Pyramid Pooling (ASPP) module to cope with different object scales. However, they do not carry enough global context information to handle large objects. In contrast to the previous methods, the proposed GLNet incorporates global and local context information, together with low-level feature to tackle complex scenes that contain a mixture of fine and large objects.

Recently, DANet [21] reached a new state-of-the-art performance level of 81.5 on the Cityscapes dataset. However, DANet relies on extra strategies such as data augmentation, multiple feature maps of different grid sizes, and segmentation map fusion to further improve its performance [21]. Such improvement strategies can also be used in other methods. Therefore, DANet has not been included here for comparison. Without the extra steps, DANet achieved a mean IoU of 77.57, which is lower than our score.

**Figure 9:**
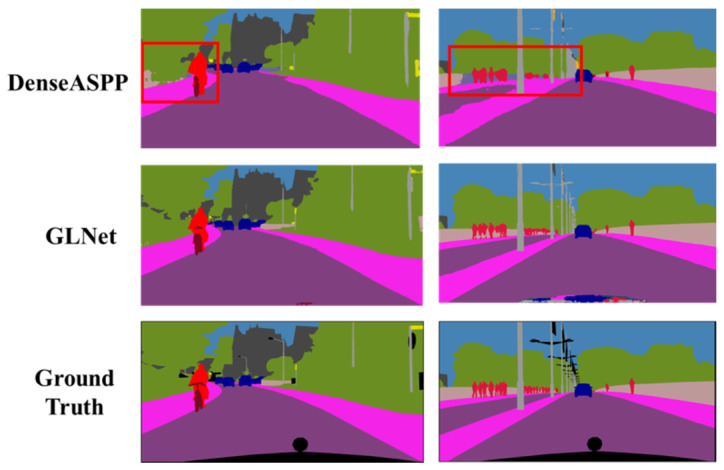
Visualization results of DenseASPP and GLNet on Cityscapes val. set.

The main difference between the proposed GLNet and existing segmentation methods is that GLNet explicitly models global contextual information, local contextual information, and low-level features in a single network and systematically combines the information for semantic segmentation of complex street view scenes. The benefit of incorporating all three pieces of information in one network enables GLNet to simultaneously dealing with large objects and background, and paying attention to fine details. Figure 9 shows visualization results of DenseASPP and GLNet, which are the top two performers in Table 4. Although the performance improvement of GLNet over DenseASPP is incremental in terms of mean IoU, it can be seen from Figure 9 that GLNet generates much better visualization results compared to DenseASPP. GLNet was able to segment large objects such as sidewalk and vegetation as well as small objects such as pedestrians and light poles equally well, whereas DenseASPP produced unsatisfactory results.

#### 4.3.2. Network Size and Inference Speed

Network size in terms of number of parameters and inference speed in frame per second (FPS) of the proposed GLNet and several other methods are listed in Table 6. The tests were running on NVIDIA GeForce GTX 1080 with Tensorflow Cuda 9.0. The test images were 1024 × 2048 raw images from the Cityscapes validation set. It can be seen from Table 6 that the number of parameters of the GLNet is slightly fewer than the DeepLab methods [6,19], and it is much lower than FCN [3] and PSPNet [18]. The inference speed of GLNet is comparable to DeepLab and PSPNet with 1.24 FPS. The difference in inference speed is partially owing to the fact that different backbone networks are used in different models. For instance, Xception-65 is used as the backbone in GLNet while DeepLab uses ResNet-101 as the backbone network.

## 5. Conclusions

We have proposed the GLNet which integrates global spatial information and dense local multi-scale context information in a single model for semantic segmentation of complex street view scenes. The global context module captures semantic of spatial interdependencies whereas the local context module extracts dense multi-scale features, and the output of the global-local module is fused with low-level features to recover fine scene details. Experimental results on the Cityscapes dataset have demonstrated superior performance of the proposed GLNet over existing state-of-the-art methods. This study highlights the importance of incorporating both global and local contextual information in image semantic segmentation. We hope this insight can contribute to future semantic segmentation works.

## Figures and Tables

**Table 1:** Performance evaluation on Cityscapes val. set.

BaseNet	ASPP	Local	Global	SE	Mean IoU%
Xception-65	✓				77.1%
Xception-65		✓			77.6%
Xception-65		✓		✓	78.4%
Xception-65		✓	✓	✓	80.1%

Note: ASPP denotes ASPP module [19], Local represents local module, Global represents global module, and SE implies SE block [24].

**Table 2:** Performance on Cityscapes val. set of applying different methods in the global module.

BaseNet	Non-Local	CBAM	Pyramid Pooling	Mean IoU%
Xception-65			✓	70.3%
Xception-65		✓		78.9%
Xception-65	✓			80.1%

**Table 3:** Performance on Cityscapes val. set using concatenation operation versus fusion operation for combining features.

BaseNet	Concatenation	Fusion	Mean IoU%
Xception-65	✓		79.9%
Xception-65		✓	80.1%

**Table 4:** Performance comparison on Cityscapes test set.

Method	Mean IoU	Road	Sidewalk	Building	Wall	Fence	Pole	Traffic light	Traffic sign	Vegetation	Terrain	Sky	Person	Rider	Car	Truck	Bus	Train	Motorcycle	Bicycle
FCN-8s [3]	65.3	97.4	78.4	89.2	34.9	44.2	47.4	60.1	65.0	91.4	69.3	93.7	77.1	51.4	92.6	35.3	48.6	46.5	51.6	66.8
DeepLab-v2 [6]	70.4	97.9	81.3	90.3	48.8	47.4	49.6	57.9	67.3	91.9	69.4	94.2	79.8	59.8	93.7	56.5	67.5	57.5	57.7	68.8
RefineNet [32]	73.6	98.2	83.3	91.3	47.8	50.4	56.1	66.9	71.3	92.3	70.3	94.8	80.9	63.3	94.5	64.6	76.1	64.3	62.2	70
DUC [33]	77.6	98.5	85.5	92.8	58.6	55.5	65	73.5	77.9	93.3	72	95.2	84.8	68.5	95.4	70.9	78.8	68.7	65.9	73.8
ResNet-38 [34]	78.4	98.5	85.7	93.1	55.5	59.1	67.1	74.8	78.7	**93.7**	**72.6**	95.5	86.6	69.2	95.7	64.5	78.8	74.1	69	76.7
PSPNet [18]	78.4	98.6	86.2	92.9	50.8	58.8	64.0	**75.6**	79.0	93.4	72.3	95.4	86.5	71.3	95.9	68.2	79.5	73.8	69.5	77.2
DenseASPP [12]	80.6	**98.7**	**87.1**	**93.4**	**60.7**	**62.7**	65.6	74.6	78.5	93.6	72.5	95.4	86.2	**71.9**	**96.0**	**78.0**	90.3	80.7	69.7	76.8
GLNet	**80.8**	**98.7**	86.7	**93.4**	56.9	60.5	**68.3**	75.5	**79.8**	**93.7**	**72.6**	**95.9**	**87.0**	71.6	**96.0**	73.5	**90.5**	**85.7**	**71.1**	**77.3**

**Table 5:** Performance comparison on Cityscapes val set.

Method	Mean IoU	Road	Sidewalk	Building	Wall	Fence	Pole	Traffic light	Traffic sign	Vegetation	Terrain	Sky	Person	Rider	Car	Truck	Bus	Train	Motorcycle	Bicycle
FCN-8s [3]	57.3	93.5	75.7	87.2	33.7	41.7	36.4	40.5	57.1	89.0	52.7	91.8	64.3	29.9	89.2	34.2	56.4	34.0	19.7	62.2
ICNet [10]	67.2	97.3	79.3	89.5	49.1	52.3	46.3	48.2	61.0	90.3	58.4	93.5	69.9	43.5	91.3	64.3	75.3	58.6	43.7	65.2
DeepLab-v2 [6]	69.0	96.7	76.7	89.4	46.2	49.3	43.6	55.0	64.8	89.5	56.0	91.6	73.3	53.2	90.8	62.3	79.6	65.8	58.0	70.2
PSPNet [18]	76.5	98.0	84.4	91.7	57.8	62.0	54.6	67.4	75.2	91.4	63.2	93.4	79.1	60.6	94.4	77.2	84.6	79.4	63.3	75.1
DeepLab-v3+ [19]	77.1	98.2	85.1	92.6	56.4	61.9	65.5	68.6	78.8	92.5	61.9	95.1	81.8	61.9	94.9	72.6	84.6	71.3	64.0	77.1
DenseASPP [12]	79.5	**98.6**	**87.0**	**93.2**	**59.9**	**63.3**	64.2	71.4	80.4	**93.1**	**64.6**	94.9	81.8	63.8	**95.6**	**84.0**	**90.8**	79.9	66.4	78.1
GLNet	**80.1**	98.4	86.7	93.1	59.5	62.7	**68.4**	**73.0**	**81.7**	92.9	64.4	**95.3**	**84.0**	**65.4**	95.3	82.6	90.6	**81.0**	**67.9**	**79.7**

**Table 6:** Network size and inference speed.

Method	Params	FPS
FCN-8s [3]	134.4 M	2.70
DeepLab-v2 [6]	43.9 M	1.38
PSPNet [18]	67.6 M	1.13
ICNet [10]	6.7 M	5.58
DeepLab-v3+ [19]	41.2 M	2.08
GLNet	39.8 M	1.24
